# Influence of mycotoxin zearalenone and its derivatives (alpha and beta zearalenol) on apoptosis and proliferation of cultured granulosa cells from equine ovaries

**DOI:** 10.1186/1477-7827-4-62

**Published:** 2006-11-30

**Authors:** Fiorenza Minervini, Alessandra Giannoccaro, Francesca Fornelli, Maria Elena Dell'Aquila, Paolo Minoia, Angelo Visconti

**Affiliations:** 1 Institute of Sciences of Food Production (ISPA), National Research Council (CNR), Via Amendola 122/O, 70124 Bari, Italy; 2 Department of Animal Production, University of Bari, Strada Provinciale per Casamassima Km 3, 70010 Valenzano Bari, Italy; 3 Deceased

## Abstract

**Background:**

The mycotoxin zearalenone (ZEA) and its derivatives, alpha and beta-zearalenol (alpha and beta-ZOL), synthesized by genera Fusarium, often occur as contaminants in cereal grains and animal feeds. The importance of ZEA on reproductive disorders is well known in domestic animals species, particularly in swine and cattle. In the horse, limited data are available to date on the influence of dietary exposure to ZEA on reproductive health and on its in vitro effects on reproductive cells. The aim of this study was to evaluate the effects of ZEA and its derivatives, alpha and beta-ZOL, on granulosa cells (GCs) from the ovaries of cycling mares.

**Methods:**

The cell proliferation was evaluated by using the 3-(4,5-dimethylthiazol-2-yl)-2,5-diphenyltetrazolium bromide (MTT) test after 3 days exposure at different concentrations of ZEA and its derivatives (from 1 × 10-7 to 0.1 microM). The apoptosis induction was evaluated after 1 day exposure, by DNA analysis using flow cytometry.

**Results:**

An increase in cell proliferation with respect to the control was observed in the presence of ZEA at 1 × 10-3 and 1 × 10-4 microM and apoptosis was induced by all mycotoxins at different concentrations.

**Conclusion:**

The simultaneous presence of apoptosis and proliferation in GC cultures treated with zearalenones could indicate that these mycotoxins could be effective in inducing follicular atresia. These effects of zearalenones may result from both direct interaction with oestrogen-receptors as well as interaction with the enzymes 3alpha (beta)-hydroxysteroid dehydrogenase (HSD), involved in the synthesis and metabolism of endogenous steroid hormones. These cellular disturbances, described for the first time in equine GCs cultured in vitro, could be hypothesized as referred to reproductive failures of unknown ethiology in the mare.

## Background

Many different mycotoxins have been recognized and isolated from a variety of *Fusarium *moulds and some of the disease states, caused by consumption of cereals containing these toxins in domestic animals as well as in humans, have been called fusariotoxicoses. Zearalenone (ZEA) and related compounds α and β zearalenol (α and β-ZOL) and α and β zearalanol (α and β-ZAL) are synthesized by a number of species of *Fusarium *such as *F. graminearum, F. tricinctum, F. moniliforme *and *F. oxysporum *[1].

Sensitivity to the effects of mycotoxins is related to species-dependent biotransformation pathways. Zearalenone is metabolized via two pathways in hepatocytes and intestinal cells, namely conjugation with glucuronic acid and reduction to α and β-ZOL by 3 α(β)-hydroxysteroid dehydrogenase (HSD) [2-4]. These reactions show similarities to processes in steroid metabolism because HSDs catalyse oxidation/reduction reactions in the synthesis and inactivation of steroid hormones [4].

The influences of ZEA on reproductive structural and functional parameters are well recognized. Of all domestic species, swine is the most sensitive species, followed by ruminants. Birds are the most resistant species [3]. The high sensitivity of pigs to ZEA-mediated oestrogenic effects can be related to ZEA bio-activation prevalently into α-ZOL. The higher estrogenicity of α-ZOL is related to its stronger relative binding affinity to the cytoplasmic oestrogenic receptor than β-ZOL, as described in the rat uterus [5].

Of all stages of maturity, the pre-pubertal gilt is the most sensitive to ZEA [6]. The changes induced by ZEA depend on the time of administration, in relation to the oestrous cycle, and on the administered dose [6]. A vulvovaginitis syndrome in young female swine and anaestrus induction in the mature sow, have been reported [7]. In extreme cases, rectal and vaginal prolapses occurred. Several atresic follicles are present on ovaries and degeneration of oocytes occurs [8-10].

Concerning the equine species, the effects of ZEA have only been demonstrated in a few cases. A field outbreak of ZEA mycotoxicosis in horses was associated with corn screenings containing approximately 2.6 mg/Kg of ZEA [11]. Raymond et al. [12,13] reported the influence of ZEA, administered at a low dose, on performance and hematological parameters. No disturbances on reproductive functions were reported for cycling mares after exposure of 1 mg/Kg of ZEA in the feed [14]. However, these levels are also ineffective on sensitive species, such as swine [7]. The limited number of reports on ZEA-related clinical symptoms in the horse could not exclude the involvement of this feed contaminant in hypo-fertility cases. No data have been reported to date concerning the pharmaco-kinesis and metabolism of ZEA in the horse.

In relation with the high incidence of ZEA-related reproductive failures in other species [15] and considering the difficulties in conducting *in vivo *studies on reproductive influence of ZEA on system in the horse, the purpose of this paper was to investigate the effects of ZEA and its derivatives, α and β-ZOL, on granulosa cells (GCs) collected from mare ovaries during the breeding season. It has been demonstrated that mural GCs are strictly involved in the control of oocyte growth and maturational competence [16] and are a sensitive target of xenoestrogen substances [17].

Premature follicular atresia is a recognized ZEA-induced consequence associated with either natural or experimental contamination [8-10]. The aim of this work was to use flow cytometric analysis in order to evaluate the atretic process in cultured GCs after treatment with zearalenones. Analysis of DNA content by flow cytometry provides a means of looking at early apoptosis and atresia as well as cell cycle perturbation and accumulation or depletion of cells in particular phases. With this method, apoptotic cells, containing less DNA than normal diploid cells, exhibit reduced fluorescence and are recorded as a sub-G_0 _peak. This method has been reported to date in porcine, bovine, ovine and rat follicles [18-22]. Atresia consists of a dynamic equilibrium between GC division, differentiation and death and simultaneous apoptosis and mitosis processes occur [23]. For this reason, an evaluation of GC proliferation was carried out using colorimetric (MTT test) and flow cytometric methods.

## Methods

### Chemicals

Zearalenone (ZEA, purity 99%), α-zearalenol (α-ZOL, purity 98%), β-zearalenol (β-ZOL, purity 95%), Dulbecco's Modified Eagle's Medium (DMEM), Ham F-12, sodium bicarbonate, gentamycin, collagenase, dimethylsulphoxide (DMSO), Trypan blue solution, foetal bovine serum (FBS), Propidium Iodide solution (PI), trypsin-EDTA solution and MTT were all purchased from Sigma-Aldrich (Italy). 2-Propanol was purchased from Baker (Holland), Ficoll-Paque Plus from Pharmacia Biotech, Uppsala (Sweden). Nonided P 40 substitute solution was purchased from Fluka (Italy)

### Collection of GCs from mare ovaries

The study was performed during the breeding season (2004–2005). Ovaries of cyclic mares at unknown stages of the oestrous cycle were obtained from an abattoir 30 Km from the laboratory. Ovaries (n = 102) were placed in physiological saline (9 g NaCl containing 40 mg/L gentamycin sulphate) within 30 min of slaughter and were transported to the laboratory in thermal containers set at 30°C (1 h transport). All antral (subordinate) follicles (0.5 to 2.5 cm) that were visible on the ovarian surface were opened with a scalpel blade and the GC layer was scraped with a curette [24]. Granulosa cells were flushed from the curette into individual Petri dishes using HEPES-buffered tissue culture medium (medium 199, Gibco BRL) supplemented with 10% FBS. The time between follicle scraping and starting of GC cultures was less than 2 h and the total time between slaughter and culture ranged between 3 and 5 h.

### GC cultures

After collection as pools from mare ovaries, GCs were washed twice in serum-free medium (DMEM\ Ham F-12 with 0.12 mM gentamycin, 2.0 mM glutamine and 38.5 mM sodium bicarbonate), recovered by centrifugation (5 min at 200 × *g*) and re-suspended in the same medium containing 1.25 mg/ml of collagenase to disperse clumping of the cells [25]. Then, the cells were layered onto 3 ml of Ficoll-Paque Plus (Pharmacia Biotech, Uppsala, Sweden) and centrifuged (10 min, 1500 × *g*, 4°C). Finally, cells were re-suspended in medium containing 10% FBS. Cell viability was determined by the Trypan blue dye exclusion test. In 15 experiments the viability ranged from 30 to 65% with a mean value of 40%. The GC pools were used to evaluate the cellular proliferation by the MTT test and the apoptosis induction by DNA analysis with flow cytometry.

### Preparation of test substances

The mycotoxins were solubilized as stock solution at a concentration of 10 mM in DMSO and then diluted in a 1:1 (v\v) mixture of DMEM and Ham F-12, containing 0.12 mM gentamycin, 2.0 mM glutamine, 38.5 mM sodium bicarbonate and 10% FBS. Mycotoxin serial dilutions (1:10) were prepared at concentrations ranging from 1 × 10^-7 ^to 0.1 μM. The concentration range used in this study corresponded to the active level (25 ng/ml) on GCs reported in bitch [26].

### Evaluation of cell proliferation by MTT test

At the beginning of each experiment, GCs were seeded in 96 multi-well plates (Iwaki, Japan) at 3 × 10^4 ^cells/well in 200 μl of medium containing 10% FBS. The cells were allowed to attach for 24 h, the medium was changed with fresh medium and each concentration of test compound was added in triplicate wells. A negative control (without mycotoxin) was included in each plate. After 3 days of incubation, cell proliferation was measured using the MTT test, as described previously [27]. The cleavage of 3-(4,5-dimethylthiazol-2-yl)-2,5-diphenyltetrazolium bromide (MTT) to a blue coloured product (formazan), which is indicative of mitochondrial succinate-dehydrogenase activity in viable cells, was quantified by spectrophotometric analysis. Absorbance was measured at 580 nm in a plate reader (ELISA Reader Multiskan MS Plus MK II Labsystem, Finland). Four experiments were performed for each mycotoxin subtype and dosage regime.

### DNA analysis by flow cytometry

The analysis of DNA by flow cytometry was performed following the method described by different authors [18-22]. At the beginning of each experiment, GCs were seeded in 24 multi-well plates (Iwaki, Japan) at 5 × 10^6^cells/well in 1 ml of medium and each concentration of test compound was added in duplicate wells. A negative control (without mycotoxin) was included in each plate. After 24 hr of incubation, cells were detached by trypsinization and the pellets were obtained by centrifugation (5 min at 200 × *g*). Cells were fixed in 96% cold ethanol (-20°C) and in cold saline solution containing 1.10 g\l glucose, 8 g\L NaCl, 0.40 g\L KCl, 0.20 g\L Na_2_HPO_4_, 0.15 g\L KH_2_PO_4 _and 0.20 g\L EDTA. Subsequently, cells were centrifuged at 300 × g for 5 min, re-suspended in 0.5 ml of cold PBS at 4°C and stained for at least 15 min with 25 μg/ml PI in 0.1% citrate sodium, RNase (1 mg/ml) and Nonided P 40 substitute solution (1%). Cells were then analysed by FACSCalibur flow cytometry (Becton Dickinson). Propidium iodide fluorescence was obtained using linear (FL-2 584–642 mm) amplification. At least 10,000 events were recorded for each sample. Cell cycle analysis was calculated by rectangular fitting (MODIFIT software, Becton-Dickinson, Milan, Italy), using 1024 channels which produced histograms with a single G_0_/G_1 _peak at channel 200 when DNA was diploid, an S-peak between channels 200 and 400 when DNA was replicating, a G_2_/M peak at channels 400 when DNA was tetraploid and a sub-G_0 _peak, between 100 and 200 when DNA was hypodiploid or damaged. The small coefficient of variation (CV) obtained in this study was the result of the high resolution achieved by proper alignment. Four independent experiments were performed for each mycotoxin treatment regime.

### Statistical analysis

All statistical analyses were performed using the GraphPad Instat Software version 2.03 (Sigma, Italy). Statistical differences between mean values and the control were evaluated by analysis of variance (ANOVA) followed by the Dunn multiple comparison post test.

## Results

### Cell proliferation

**Figure 1:**
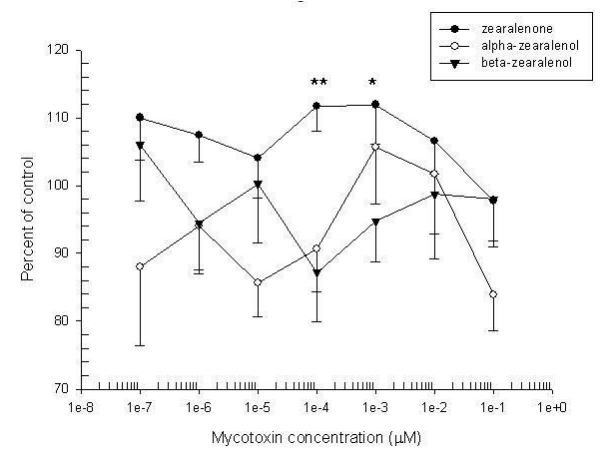
Equine granulosa cell proliferation by MTT test after 72 hours. Data represent mean ± SEM of four experiments (each concentration is tested in triplicate). * p < 0.05 and ** p < 0.01 significantly different from control values evaluated by ANOVA followed by the Dunn multiple comparison post test.

At levels of 1 × 10^-4 ^and 1 × 10^-3 ^μM, ZEA induced a statistically significant increase in cell proliferation compared to the control (p < 0.01 and 0.05, respectively; Fig 1). The tested concentrations of α and β-ZOL did not induce increases in cell proliferation. High doses of ZEA and its metabolites demonstrated a trend towards down-regulation of GC proliferation indices.

### DNA analysis

**Figure 2:**
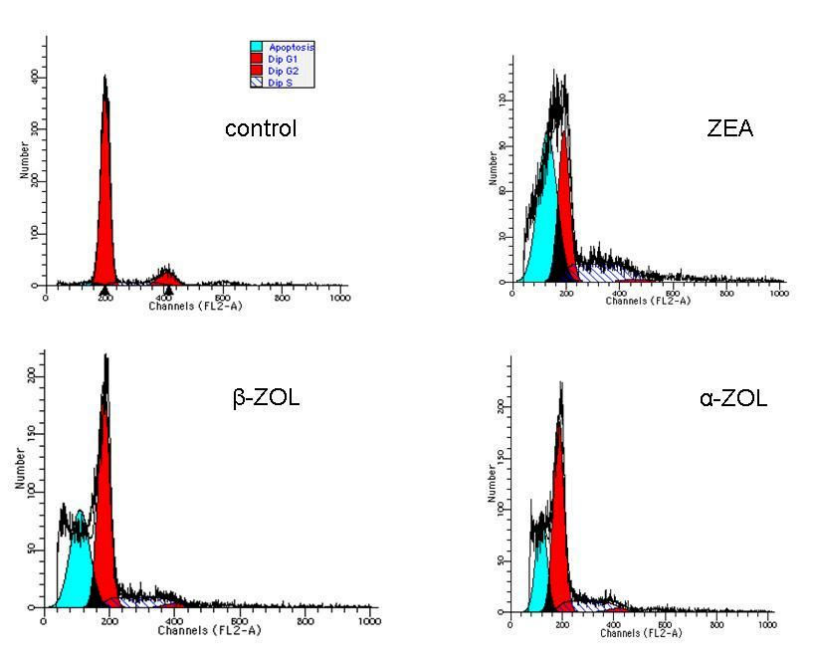
A representative DNA analysis of equine granulosa cells exposed to zearalenone and its derivatives using flow cytometry.

As observed in a representative experiment (Fig. 2), all mycotoxins induced apoptosis, evaluated as a sub-G_0 _peak, on equine GCs at different concentration levels. Alpha-zearalenol was more active and induced apoptosis at 1 × 10^-3 ^μM (28% of the sub-G_0 _peak compared to 4% observed in the control sample), following by ZEA at 1 × 10^-2 ^μM (50% of sub-G_0 _peak) and β-ZOL at 1 × 10^-1 ^μM (37% of sub-G_0 _peak).

**Figure 3:**
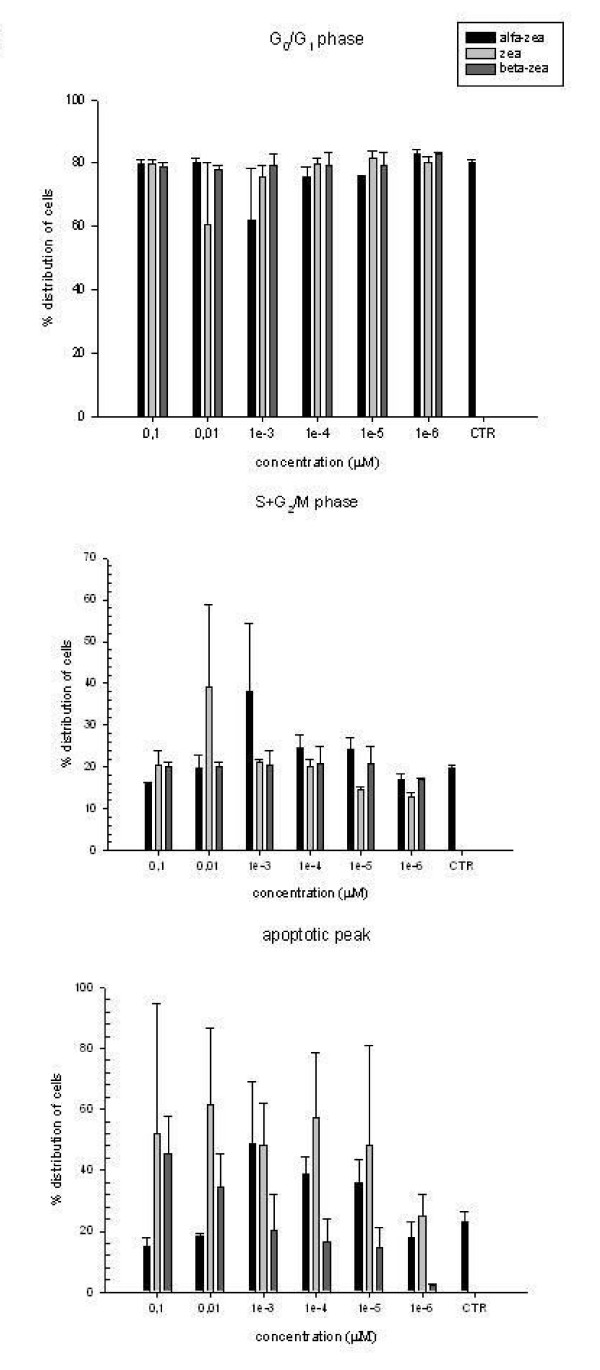
Modification of cell cycle of equine granulosa cells following exposure to zearalenones. Data represent mean ± standard deviation of four experiments.

The percentages of GCs in G_0_/G_1 _(cells in the stationary phase), S+G_2_/M (cells in proliferation) and sub-G_0 _(apoptotic cells) phases found in all experiments are shown in Fig 3. No statistical difference was observed following treatment with zearalenones and a high variability between experiments was found. For α-ZOL, a concomitant 2 fold increase in the sub-G_0 _and S+G_2_/M peaks was observed at 1 × 10^-3 ^μM. The apoptotic process was induced, at low intensity (1.5 fold of increase in the sub-G_0 _peak with respect to the control), up to 1 × 10^-5 ^μM. A 23% reduction of the G_0_/G_1 _phase was found after exposure to 1 × 10^-3 ^μM of α-ZOL (Fig. 3). At lower concentrations, the cell cycle was similar to the control. Concerning ZEA, at 1 × 10^-2 ^μM the sub-G_0 _and S+G_2_/M phases increased simultaneously 2.7 and 2 fold, respectively. A two fold increase in the sub-G_0 _peak continued until 1 × 10^-5 ^μM of ZEA. A 24% reduction in the G_0_/G_1 _peak was observed at 1 × 10^-2 ^μM of ZEA (Fig 3). Other concentrations of ZEA did not modify the cell cycle of GCs compared to the control. Concerning β-ZOL, the maximum increase (2 fold) in the sub-G_0 _peak was observed at the highest concentration and continued, at low intensity (1.2 fold of increase), up to 1 × 10^-2 ^μM. No variations to other phases of the cell cycle were observed in the presence of β-ZOL (Fig 3). Other concentrations of β-ZOL did not modify the cell cycle of GCs compared to the control.

## Discussion

An increase in cell proliferation of equine GCs was observed using the MTT test in the presence of 1 × 10^-4 ^and 1 × 10^-3 ^μM of ZEA. High doses of ZEA and its metabolites demonstrated a trend towards down-regulation of GC proliferation indices. This increased proliferation could be the consequence both of the reduction of ZEA into α-ZOL and the modification of ovarian steroidogenesis with accumulation of the active hormonal components related to the involvement of 3α(β)-HSD, enzymes present in the ovary and GCs [3,29]. The HSDs have also been claimed to be important factors in ovarian follicular development and their inhibition may promote follicular atresia, since abnormalities in the androgen, progestin and oestrogen metabolism can lead to ovarian dysfunction [3,28]. Zearalenone and its derivatives induced apoptotic processes within equine CGs at different concentrations. The mean intensity of apoptosis induced by ZEA was greater than that caused by α-ZOL, probably derived from the combined effect exerted by both mycotoxins metabolized by HSD in GCs, as reported previously [29]. Beta-zearalenol was less active than other mycotoxins because it induced apoptosis only at the highest concentration.

In this study, ZEA and its derivatives did not induce statistically different modifications of cell distribution in different phases of the cell cycle. However, it should be noted a trend in growth increasing, evaluated as S+G_2_/M proliferation peak, that was affected by 1 × 10^-2 ^and 1 × 10^-3 ^μM of ZEA and α-ZOL, respectively. The observed variability could be related to the different levels of HSDs in GS cultured in each experiment, as reported by other authors [30]. In addition, seasonal (light, temperature), environmental (stress, diet) and hormonal factors (including the enzymatic contents) as well as the holding time and temperature can influence the variability observed during experiments carried out with equine GCs [31]. Though no statistical differences were found in our experiments, simultaneous increases in the sub-G_0 _and S+G_2_/M peaks indicate a simultaneous occurrence of apoptosis and the proliferation process after exposure with ZEA and α-ZOL. This dynamic equilibrium between division, differentiation and death was found by Pedersen et al. [32] in atretic follicles. The simultaneous occurrence of proliferation and apoptosis was studied by Quirk et al [33,34] in bovine GCs. These authors reported that oestradiol, an important intraovarian growth, differentiation and survival factor, increases the progression from G_0_/G_1 _to S phase, the percentage of cells in S phase and, consequently, the percentage of DNA synthesizing cells and that this cell cycle progression is mediated by the oestrogen receptor [33]. Granulosa cells may be most susceptible to apoptosis at the transition from G_1 _to S phase [34]. We hypothesize that zearalenones, oestrogen-like substances, can act with a similar mechanism on equine GCs and can induce proliferation and apoptotic process.

Follicular degeneration, or atresia, is the result of an apoptotic process defined as a sequence of programmed intracellular events leading to cell death [20]. The first signs of atresia are evidenced as the degeneration of the antral GCs, whereas the oocyte is affected in the last stages of atresia [35,36]. Various techniques have been adopted to measure the degree of follicular degeneration in ovaries. Few reports are available to date on apoptosis of GCs in horse. Okolski et al. [37] evaluated equine GCs for the presence of nuclear fragments they termed atretic bodies. The presence of these bodies was correlated with reduced oestrogen levels in the follicular fluid but was not correlated with the proportion of oocytes that matured *in vitro *[37]. Pedersen et al. [32], the first group using DNA laddering to study apoptosis in the mares, found that small follicles were more likely than large follicles to be apoptotic. These authors reported that the histological method used for determining the presence of atresia was not reliable because atretic GCs were not uniformly distributed but were observed in focal points, within areas of healthy GCs [32]. Albrizio et al. [38] investigated follicular wall apoptosis in the mare by comparing DNA laddering and the incidence of apoptotic bodies (AB) analyzed by Hoechst 33258 staining. These authors found that high rates (80%) of typical masses of AB were observed in GCs of small follicles from anoestrous mares and, with a lower incidence, in pools from regressing follicles collected from the ovaries of transitional, oestrous and dioestrous mares (30–40%). Large pre-ovulatory follicles were totally lacking AB. Correspondingly, the presence of DNA fragments forming the characteristic banding pattern (ladder) of apoptosis was revealed in the GCs from anoestrous mares; these fragments were not detected in the GCs from large pre-ovulatory follicles while they had an intermediate incidence in the follicles from ovaries of transitional and dioestrous mares. Dell'Aquila et al. [39] reported that GC apoptosis in the mare is related to cumulus expansion and to an increase in oocyte meiotic competence but has no effect on the proportion of meiotically competent oocytes that activate after ICSI. Subsequently, Pedersen et al. [40] detected GC apoptosis by comparing two biochemical methods, 3'-end labelling and staining with ethidium bromide then microscopic examination of GC morphology, with the established method of detecting atresia by histology. These authors concluded that the more parameters used in conjunction with each other, the more accurate was the analysis of the state of the follicle. Many studies have shown that GCs with hypodiploid DNA content are apoptotic, may represent a biochemical marker for atretic follicles in several animal species and that this method is sensitive enough to detect apoptosis in its very early stages [18-22]. Granulosa cells that have initiated chromatin cleavage are definitively non-functional and flow cytometry enables the rapid detection of cells that are undergoing the early events of apoptosis, as previously reported in porcine, bovine, ovine and rat follicles [18-22]. In our experimental conditions, flow cytometry was effective and rapid for evaluating the in vitro effects of the mycotoxin on GC apoptosis in the mare. This is the first report on the use of flow cytometry to study the atretic process in mare cultured GCs.

A "mare reproductive loss syndrome (MRLS)", which occurred in Kentucky in 2001, was characterized by non etiologically defined cases of early and late-term abortions [41]. Different etiological agents were thought to be responsible for this pathology, such as ergot alkaloids, phytoestrogens or mycotoxins, caterpillar and *Actinobabacillus *spp[41]. The possible role of the mycotoxin ZEA as a cause of MRLS has been reviewed by Newman, 2003 [42] and Newman and Raymond, 2005 [43]. Our data integrate previous *in vitro *observations conducted on both the germinal and somatic compartments of ovarian follicles in animal models as well as in humans. In sows, ZEA and its hydroxylated metabolites (α and β-ZOL) exerted negative effects on oocyte maturation [44] and inhibited the FSH-, forskolin- and pregnenolone-stimulated progesterone synthesis in GCs [45]. In cattle, ZEA and its derivatives inhibited the maturation of oocytes to metaphase II and high levels of 17 β-estradiol production by mural GCs were found [46]. The negative influence of ZEA was reported by Skorska-Wyswynska et al. [26] on cellular viability and morphology of cultured granulosa and internal theca cells of ovarian follicles in bitches. In human granulosa-luteal cells, ZEA was a potent inhibitor of aromatase/17 β HSD up to 10^-7 ^M with consequent inhibition of the conversion of androstenedione to oestradiol [17]. No *in vitro *studies have been previously carried out in the mare reproductive tract.

## Conclusion

Equine GCs are sensitive to the effects of zearalenones, especially to α-ZOL and ZEA. This rank of activity was in agreement to that observed *in vitro *for other farm animals [45,46]. This sensitivity of equine GCs to oestrogenic effects induced by ZEA and its derivatives emphasizes the importance of ZEA in reproductive disorders in this species. Studies are in progress to deepen understanding the mode of action of zearalenones on apoptosis and cell proliferation in GCs and the influence of zearalenones on reproductive function in the mare.

## Authors' contributions

FM = the author responsible for conceiving the research project, interpretation of data and involved in drafting the manuscript especially concerning the mycotoxin field.

AG = the author responsible for designing and performing the proliferation analysis and statistical analysis

FF = the author responsible for designing and performing the DNA analysis and statistical analysis.

MED = the author responsible for the isolation of granulosa cells and involved in drafting the manuscript especially concerning the section on equine reproductive biology and biotechnology

PM = The author involved in critically revising the manuscript especially concerning equine obstetrical and gynaecological details. He died during preparation of the manuscript and we dedicate this article to his long-standing contribution to the animal reproduction field.

AV = the author involved in critically revising the manuscript for important intellectual content and the supervisor of the research group.
